# Stage-Specific Class I Nucleases of *Leishmania* Play Important Roles in Parasite Infection and Survival

**DOI:** 10.3389/fcimb.2021.769933

**Published:** 2021-10-15

**Authors:** Anita Leocadio Freitas-Mesquita, José Roberto Meyer-Fernandes

**Affiliations:** ^1^Instituto de Bioquímica Médica Leopoldo De Meis, Universidade Federal do Rio de Janeiro, Rio de Janeiro, Brazil; ^2^Instituto Nacional de Ciência e Tecnologia em Biologia Estrutural e Bioimagem, Rio de Janeiro, Brazil

**Keywords:** class I nucleases, *Leishmania* lifecycle, *Leishmania spp*., parasite infection, parasite survival

## Abstract

Protozoans of the genus *Leishmania* are the causative agents of an important neglected tropical disease referred to as leishmaniasis. During their lifecycle, the parasites can colonize the alimentary tract of the sand fly vector and the parasitophorous vacuole of the mammalian host, differentiating into distinct stages. Motile promastigotes are found in the sand fly vector and are transmitted to the mammalian host during the insect blood meal. Once in the vertebrate host, the parasites differentiate into amastigotes and multiply inside macrophages. To successfully establish infection in mammalian hosts, *Leishmania* parasites exhibit various strategies to impair the microbicidal power of the host immune system. In this context, stage-specific class I nucleases play different and important roles related to parasite growth, survival and development. Promastigotes express 3’-nucleotidase/nuclease (3’-NT/NU), an ectoenzyme that can promote parasite escape from neutrophil extracellular traps (NET)-mediated death through extracellular DNA hydrolysis and increase *Leishmania*-macrophage interactions due to extracellular adenosine generation. Amastigotes express secreted nuclease activity during the course of human infection that may be involved in the purine salvage pathway and can mobilize extracellular nucleic acids available far from the parasite. Another nuclease expressed in amastigotes (P4/LmC1N) is located in the endoplasmic reticulum of the parasite and may be involved in mRNA stability and DNA repair. Homologs of this class I nuclease can induce protection against infection by eliciting a T helper 1-like immune response. These immunogenic properties render these nucleases good targets for the development of vaccines against leishmaniasis, mainly because amastigotes are the form responsible for the development and progression of the disease. The present review aims to present and discuss the roles played by different class I nucleases during the *Leishmania* lifecycle, especially regarding the establishment of mammalian host infection.

## Introduction

*Leishmania* spp. are trypanosomatid parasites that infect humans and other mammalian hosts, causing one of the most significant of neglected tropical diseases (Chang, 1983). Leishmaniasis affects more than 350 million people worldwide and is the major insect-borne disease in developing countries (Vannier-Santos et al., 2002). The manifestations of the disease include cutaneous, mucocutaneous, diffuse cutaneous, and visceral leishmaniasis, which is the most severe form of the disease. These different manifestations are related to the complex interaction between the infecting species and the host immune response (Pace, 2014).

During their lifecycle, the parasites alternate between the promastigote form that resides in the alimentary tract of the sandfly vector and the amastigote form that is found inside the parasitophorous vacuoles of mammalian host mononuclear phagocytes. Promastigotes are transmitted from the sand fly vector to the mammalian host during blood meal consumption (Vannier-Santos et al., 2002). Since their inoculation, parasites face diverse hostile microenvironments and present various strategies to impair the microbicidal power of the host immune system and survive and proliferate during the course of infection (Podinovskaia and Descoteaux, 2015). In this context, the current review aims to present and discuss the roles played by different class I nucleases during the *Leishmania* lifecycle, especially regarding the establishment of mammalian host infection.

Class I nucleases hydrolyze nucleic acids with RNA as their main substrate and present nucleotidase activity that can hydrolyze the phosphate group at the 3’ position of 3’-monophosphorylated nucleotides. Several members of the class I nuclease family have been identified in plants, protozoa and fungi (Wilson, 1982; Desai and Shankar, 2003). The P1 nuclease of *Penicillium citrinum*, which is considered one of the archetypes of class I nucleases, has been studied for its three-dimensional structure in more detail. Crystallographic assays have revealed the presence of three coordinated Zn^2+^ ions that delimit the active site of the enzyme. The proposed mechanism for catalysis involves a nucleophilic attack of Zn^2+^ activated by a water molecule (Volbeda et al., 1991; Romier et al., 1998). The sequential alignment of class I nuclease family members revealed the existence of five highly conserved regions, four of which have one or more histidine residues and are likely to be involved in the binding of Zn^2+^ ions (Yamage et al., 2000).

Throughout this review, we provide an overview of the major biochemical properties of the class I nucleases identified in several *Leishmania* species, as well as their differential expression throughout the parasite lifecycle. We also discuss the physiological roles that have been attributed to these enzymes, highlighting their potential uses in leishmaniasis chemotherapy and prophylaxis.

## Promastigote Stage-Specific Class I Nuclease

### 3’-NT/NU

To successfully establish infection in the mammalian host, *Leishmania* parasites must impair the microbicidal repertoire of neutrophils and macrophages. One of the strategies for neutrophil-mediated killing is the release of a lattice composed of DNA associated with histones and granular and cytoplasmic proteins named neutrophil extracellular traps (NETs). NETs can ensnare and kill microorganisms, preventing parasitic infection (Brinkmann et al., 2004; Guimarães-Costa et al., 2012). For several microorganisms, the expression of secreted or membrane-bound nucleases has been reported as a strategy to escape the toxic effects promoted by NETs (Sumby et al., 2005; Berends et al., 2010; Seper et al., 2013; Thammavongsa et al., 2013; Afonso et al., 2021). The bifunctional enzyme 3’-nucleotidase/nuclease (3’-NT/NU), a unique class I nuclease present in several *Leishmania* species, has been considered an important factor for parasite escape from NET-mediated death through the hydrolysis of extracellular DNA (Guimarães-Costa et al., 2014; Freitas-Mesquita et al., 2019).

3’-NT/NU was first described in *Leishmania donovani* parasites (Gottlieb and Dwyer, 1983). Acid phosphatase, 5’-nucleotidase, and 3’-nucleotidase activities were observed in the surface membrane fraction isolated from *L. donovani* promastigotes. Based on biochemical properties, primarily differential sensitivity to inhibitors, these activities were shown to correspond to different enzymes (Gottlieb and Dwyer, 1983). Posterior studies performed with several *Leishmania* species have shown that 3’-NT/NU presents stage-specific expression. While procyclic and metacyclic promastigotes express the enzyme, no expression is observed in amastigotes (Sopwith et al., 2002; Lakhal-Naouar et al., 2008).

Due to similarities in biochemical parameters and structure, the enzyme 3’-NT/NU was classified as a member of the class I nuclease family (Neubert and Gottlieb, 1990). Sequence analyses of the gene encoding 3’-NT/NU in *L. donovani* (Debrabant et al., 1995; Debrabant et al., 2000), *Leishmania mexicana* (Sopwith et al., 2002), *Leishmania major* (Lakhal-Naouar et al., 2008), and *Leishmania amazonensis* (Paletta-Silva et al., 2011) have confirmed the presence of the five highly conserved regions associated with the class I nuclease family. 3’-NT/NU is a unique class I nuclease characterized as a cell surface membrane-anchored protein (Debrabant et al., 1995). In accordance with its previously observed ectoactivity, analysis of functional domains has shown the presence of an N-terminal signal peptide that targets the enzyme to the endoplasmic reticulum and a C-terminal transmembrane domain that anchors the enzyme to the parasite surface (Debrabant et al., 2000; Yamage et al., 2000).

In terms of biochemical analyses, the 3’-nucleotidase activity of 3’-NT/NU has been vastly studied, with reports in several species, including *L. donovani* (Gottlieb and Dwyer, 1983), *L. mexicana* (Hassan and Coombs, 1987), *L. major* (Lakhal-Naouar et al., 2008), *Leishmania chagasi* (Vieira et al., 2011), and *L. amazonensis* (Paletta-Silva et al., 2011). However, few studies regarding the nuclease activity of 3’-NT/NU, which is referred to as ecto-nuclease activity, are available. The first study to perform full biochemical characterization of ectonuclease activity in *Leishmania* parasites was recently carried out with *L. amazonensis* (Freitas-Mesquita et al., 2019). The biochemical parameters were determined by evaluating the hydrolysis of extracellular nucleic acids using the purified recombinant enzyme and living promastigotes. This activity was shown to be more efficient at alkaline pH values, as previously observed for the ecto-3’-nucleotidase activities of *L. donovani* (Gottlieb and Dwyer, 1983), *L. mexicana* (Hassan and Coombs, 1987), and *L. amazonensis* (Paletta-Silva et al., 2011). RNA, DNA, and different polyribonucleotides were efficiently hydrolyzed by *L. amazonensis*, which showed a preference for RNA, Poly-U, and Poly-A, which is consistent with the substrate specificity previously determined in *L. donovani* (Campbell et al., 1991).

**Figure 1:**
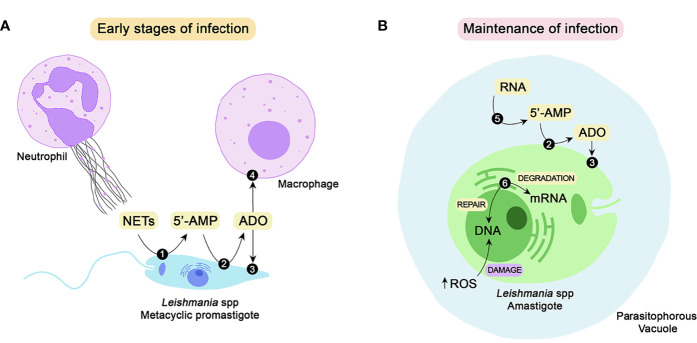
Class I nucleases of *Leishmania* spp. and their possible roles during the establishment and maintenance of parasite infection. To successfully establish infection in the mammalian host, *Leishmania* promastigotes must impair the microbicidal repertoire of neutrophils and macrophages. One of the strategies for neutrophil-mediated killing is the release of NETs. The parasite can escape from the traps by DNA hydrolysis performed by 3’-NT/NU (1), a membrane-bound class I nuclease. Hydrolysis of extracellular nucleic acids generates 5’-monophosphorylated nucleotides, including 5’-AMP, that can be converted to adenosine by the action of ecto 5’-nucleotidase (2). Adenosine can be uptaken by parasites through nucleoside transporters (3) to supply the purine salvage pathway or bind to purinergic receptors (4) of host macrophages, thus favoring parasite infection **(A)**. To maintain the infective process, the parasites must differentiate into amastigotes, which can survive and proliferate inside parasitophorous vacuoles. Amastigotes express a secreted nuclease (5) that may mobilize extracellular nucleic acids located around the parasite and convert them to nucleotides, thus contributing to the purine salvage pathway. Another class I nuclease selectively expressed by amastigotes is P4/LmC1N nuclease (6). Located at the endoplasmic reticulum, this nuclease is probably involved in gene expression through mRNA degradation. Due to its endonuclease activity, this enzyme may also promote DNA repair, subverting the eventual damage caused by the oxidative burst **(B)**. NETs, neutrophil extracellular traps; 5’-AMP, adenosine-5’-monophosphate; ADO, adenosine; RNA, ribonucleic acid; DNA, deoxyribonucleic acid; ROS, reactive oxygen species.

Trypanosomatids do not express the enzymes responsible for *de novo* synthesis of purines; therefore, they are strictly dependent on host sources. As nucleotides and nucleic acids cannot be transported across the plasma membrane, their sequential hydrolysis to nucleosides constitute an important step in the purine acquisition process (Hammond and Gutteridge, 1984; Gottlieb, 1989). Through its nucleotidase activity, 3’-NT/NU can generate extracellular nucleosides through dephosphorylation of 3’-monophosphorylated nucleotides. Moreover, hydrolysis of extracellular nucleic acids generates 5’-monophosphorylated nucleotides that can be converted to nucleosides by the action of ecto 5’-nucleotidase, another ectoenzyme present in the plasma membranes of several trypanosomatids (Gottlieb, 1989), as summarized in **Figure 1A**.

Studies with *L. donovani* and *L. amazonensis* have shown that the expression and activity of 3’-NT/NU are stimulated when parasites face purine deprivation during growth, corroborating their involvement in the purine salvage pathway (Gottlieb, 1985; Freitas-Mesquita et al., 2019). The starvation of inorganic phosphate (P_i_), which is one of the products of the reactions catalyzed by 3’-NT/NU, can also positively modulate ecto-3’-nucleotidase activity, as observed in *L. donovani* (Sacci et al., 1990) and *L. chagasi* (Vieira et al., 2011).

The role of 3’-NT/NU in the significant generation of extracellular adenosine, which is promoted by 3’-AMP hydrolysis or even by the sequential hydrolysis of nucleic acids to nucleosides, is also important from an immunological perspective. A adenosine can interact with purinergic receptors, triggering the release of anti-inflammatory cytokines and impairing the production of proinflammatory cytokines, thus favoring the establishment of parasite infection, as shown in **Figure 1A** (Paletta-Silva and Meyer-Fernandes, 2012; Freitas-Mesquita and Meyer-Fernandes, 2014; Freitas-Mesquita and Meyer-Fernandes, 2017).

Parasite and macrophage *in vitro* interaction assays have shown that the addition of 3’-AMP to the coculture medium promotes an increase in association indices, as observed in *L. chagasi* and *L. amazonensis*. The positive modulation promoted by 3’-AMP was equivalent to that observed with the same concentration of adenosine (Paletta-Silva et al., 2011; Vieira et al., 2011). Moreover, when 3’-AMP was added together with 3’-NT/NU inhibitors, such as tetrathiomolybdate (TTM) and guanosine 5’-monophosphate (5’-GMP), the stimulatory effect was completely reverted (Paletta-Silva et al., 2011; Freitas-Mesquita et al., 2016). Taken together, these results confirm that the modulation exerted by 3’-AMP is related to its conversion to adenosine by the action of 3’-NT/NU.

Although ectonuclease activity may indirectly contribute to adenosine generation, its major biological role seems to be related to parasite escape from NETosis (**Figure 1A**). An increase in the survival rate upon interaction with neutrophils was correlated with deprivation of P_i_ and purines during the growth of *L. infantum* (Guimarães-Costa et al., 2014) and *L. amazonensis* (Freitas-Mesquita et al., 2019), respectively. As previously reported, 3’-NT/NU activity is sensitive to P_i_ and purine contents in culture medium and is increased when parasites are starved of these nutrients (Gottlieb, 1985; Sacci et al., 1990; Vieira et al., 2011). Furthermore, pretreatment of *L. infantum* parasites with TTM and 5’GMP, two 3’-NT/NU inhibitors, resulted in a decrease in NET degradation (Guimarães-Costa et al., 2014). These correlations strongly suggested the participation of 3’-NU/NU in NET hydrolysis, which was confirmed by the results obtained with the purified recombinant protein. The addition of r3’-NT/NU increased parasite survival after coculture with neutrophils and after incubation with NET-enriched supernatant. The effect promoted by r3’-NT/NU was similar to that obtained by DNase, which is known to be able to destroy NETs (Freitas-Mesquita et al., 2019).

## AMASTIGOTE STAGE-SPECIFIC CLASS I NUCLEASES

### P4/LmC1N

Several different drugs are available for the treatment of leishmaniasis, but pentavalent antimony-containing compounds remain to be used as the standard treatment, mainly in Latin America (Herwaldt and Berman, 1992; Berman, 1997; Ashutosh et al., 2007). However, an increase in therapeutic failure has been noted in the past few years due to the emergence of resistant parasites (Croft and Olliaro, 2011). In this context, the development of a vaccine against leishmaniasis is an extremely important aspect for the control of this disease, which affects millions of people worldwide. Although no effective vaccines against human leishmaniasis are currently available, hundreds of potential candidates are being studied (Singh and Sundar, 2012), including a class I nuclease selectively expressed by amastigotes of different *Leishmania* species (Kar et al., 2000; Campbell et al., 2003; Fakhraee et al., 2016).

Previous studies have shown that inoculation of living promastigotes is effective for preventing Old World cutaneous leishmaniasis (CL) (Greenblatt, 1980; Modabber, 1995). However, vaccination with virulent parasites is currently considered ethically unacceptable due to adverse reactions in susceptible individuals (Stober et al., 2006). First-generation vaccines have shown low efficacy since they are based on the use of killed parasites that do not mimic natural infection and are less immunogenic (Dunning, 2009; Fakhraee et al., 2016). On the other hand, second-generation vaccines induce protection using parasite antigens that may be obtained from native fractions purified from parasites (Rachamim and Jaffe, 1993; Borja-Cabrera et al., 2009; Moreno et al., 2014) or from recombinant bacteria or viruses carrying Leishmania antigen genes (De Oliveira et al., 2000; Smooker et al., 2004; Palatnik-de-Sousa et al., 2008; Miura et al., 2015). As amastigotes represent the parasite stage responsible for the pathology associated with leishmaniasis, amastigote-specific antigens are of particular interest for the development of an efficient vaccine (Kar et al., 2000; Farajnia et al., 2004). One of the first studies to investigate stage-specific purified antigens reported that three antigens selectively expressed amastigotes (P2, P4, and P8) to confer partial to complete protection against infection with *Leishmania pifanoi* and *L. amazonensis* in BALB/c mice (Soong et al., 1995).

To obtain more information on this potential prophylactic target, the gene encoding the P4 antigen of *L. pifanoi* was cloned and sequenced (Kar et al., 2000). Comparative analyses using DNA-derived protein sequences have revealed significant levels of identity with the 3’-NT/NU of *L. donovani* (33.7%) and the P1 zinc-dependent nuclease of *Penicillium citrinum* (20.8%), suggesting that the P4 antigen possesses nuclease activity (Kar et al., 2000). This biological property was further confirmed by biochemical assays. Using different substrates to measure the enzymatic activity of affinity-purified *L. pifanoi* P4, the protein was observed to display endo- and exonuclease activity and can hydrolyze single-stranded DNA and RNA. 3’-monophosphorylated nucleotides are also substrates for P4 nuclease, revealing the presence of phosphomonoesterase activity (Kar et al., 2000).

Despite the level of homology and the similarities in substrate specificity, P4 nuclease differs from 3’-NT/NU in several aspects (Gottlieb and Dwyer, 1981; Gottlieb and Dwyer, 1983; Debrabant et al., 1995; Kar et al., 2000). While 3’-NT/NU is an external surface membrane protein, P4 nuclease appears to have a perinuclear location. Immunofluorescence analyses showed that P4 protein colocalizes with a binding protein (BiP), a marker of the endoplasmic reticulum (Bangs et al., 1996). Notably, nuclease activities associated with the endoplasmic reticulum are involved in mRNA stability (Bandyopadhyay et al., 1990; Ross, 1996). In this context, P4 nuclease can be speculated to play a role in gene regulation and expression. Due to its endonuclease activity, the P4 protein may also be involved in nucleotide excision and repair. This property may be crucial for parasite survival in the mammalian host once inside the phagolysosome of a macrophage. *Leishmania* parasites are constantly subjected to the oxidative burst that promotes DNA damage (Kar et al., 2000). These possible biological roles are schematized in **Figure 1B**.

In addition to localization, the most remarkable difference between 3’-NT/NU and P4 nuclease is their differential expression during the parasite lifecycle. The expression of 3’-NT/NU and P4 nuclease is virtually restricted to the promastigote and amastigote stages, respectively. Northern blot analyses using RNA purified from *L. pifanoi* and *L. amazonensis* have confirmed previous reports that P4 protein is exclusively expressed by amastigotes (Soong et al., 1995; Kar et al., 2000)

Southern blot analyses have shown that homologs of the P4 gene are present in other *Leishmania* species, including *L. amazonensis*, *L. braziliensis*, *L. major* and *L. donovani* (Kar et al., 2000). A novel class I nuclease was posteriorly identified in *L. major* (LmaC1N), presenting high similarity (87%) to the P4 nuclease of *L. pifanoi* (Farajnia et al., 2004). The gene encoding LmaC1N was cloned using primers specific for conserved regions of class I nucleases of trypanosomatids, and deduced sequence analyses confirmed the existence of all five conserved regions (Farajnia et al., 2004). Similar to P4 nuclease and in contrast to 3’-NT/NU, LmaC1N was selectively expressed in amastigotes rather than promastigotes, as observed by RT–PCR and Western blotting assays (Farajnia et al., 2004).

Posteriorly, a homolog of P4 nuclease was identified in *L. infantum*, the causative agent of visceral leishmaniasis. The P4 nuclease gene was cloned, sequenced, and heterologously expressed for further characterization (Farajnia et al., 2011). Comparative sequence analyses have shown high homology to the P4 nucleases of *L. donovani*, *L. major* and *L. pifanoi*. The alignment results confirmed the existence of the five conserved domains of class I nucleases (Farajnia et al., 2011). Western blot analyses have shown that the P4 nuclease of *L. infantum* is expressed in both promastigotes and amastigotes. However, in accordance with previous studies involving this enzyme, its expression is significantly higher in amastigote parasites (Farajnia et al., 2011).

The high conservation among several *Leishmania* species, as well as the extensive expression found in the amastigote stage, renders P4 nuclease an excellent target for the development of a pan-*Leishmania* vaccine (Kar et al., 2000; Coler and Reed, 2005; Farajnia et al., 2011; Fakhraee et al., 2016). The first observations in this area showed that intraperitoneal injections of P4 antigen administered with *Corynebacterium parvum* as an adjuvant provided significant protection to BALB/c mice challenged with *L. pifanoi* promastigotes (Soong et al., 1995). The immunized mice developed smaller or no lesions, showing a significant parasite burden reduction after two weeks of infection. An increase in the levels of interferon gamma (IFN-γ) production was observed when immunized mice were stimulated with parasite antigens, suggesting that the resistance induced by P4 antigen is associated with a T helper 1 (Th1) cell-mediated immune response (Soong et al., 1995). Posteriorly, CD4^+^ T cells of P4-vaccinated mice were observed to produce not only IFN-γ but also macrophage migration inhibitory factor (MIF) and tumor necrosis factor/lymphotoxin (TNF/LT), leading to intracellular parasite destruction *in vitro* (Kar 2005).

A few years later, a DNA-based vaccine was tested using the *L. amazonensis* gene encoding the P4 nuclease associated with adjuvant constructs encoding murine interleukin-12 (IL-12) and *L. amazonensis* heat-shock protein 70 (HSP70) (Campbell et al., 2003). Both IL-12 and HSP70 have been reported to elicit Th1-type responses (Mattner et al., 1996; Wang et al., 2002). P4/IL-12-immunized BALB/c mice developed potent immune protection against *L. amazonensis* but remained susceptible to *L. major* infection. On the other hand, the P4/HSP70 vaccine only delayed the emergence of lesions in *L. amazonensis*-infected mice but was highly efficient against *L. major*, leading to a self-healing phenotype in infected mice (Campbell et al., 2003). To develop a DNA vaccine that promotes cross-protection against different *Leishmania* species, determining the optimal combination of several parasite genes and an appropriate adjuvant is crucial. Based on these results, P4 and HSP70 appear to be promising candidates for the development of a DNA-based vaccine for both New and Old World *Leishmania* species (Campbell et al., 2003).

Other studies in this field were performed based on LmaC1N protein, the P4 nuclease homolog from *L. major*. To evaluate LmaC1N as a potential human vaccine candidate, cellular immune responses to recombinant LmaC1N (rLmaC1N) were examined in individuals who had recovered from Old World cutaneous leishmaniasis (Farajnia et al., 2005). In addition to being recognized in 90% of the individuals tested, rLmaC1N was shown to elicit strong Th1-like responses characterized by high levels of IFN-γ, low levels of IL-10, and minimal IL-5 production (Farajnia et al., 2005). Later, the first report using animal models showed that the use of liposome-polycation-DNA (LPD) as an immunoadjuvant renders rLmaC1N an appropriate candidate for developing a suitable vaccine against leishmaniasis (Fakhraee et al., 2016). BALB/c mice vaccinated with rLmaC1N plus LPD nanoparticles showed delayed emergence of skin lesions as well as a delay in the spread of *L. major* from the inoculation site to the spleen. The statistically significant advantageous effects observed for rLmaC1N’s association with LPD nanoparticles compared to the effects obtained without coadministration of this adjuvant demonstrate the importance of selecting an efficient antigen delivery system for better protection (Fakhraee et al., 2016).

### LdNuc^s^

Beyond the membrane-bound 3’-NT/NU and the intracellular P4 nuclease, a secretory class I nuclease has also been identified and shown to be conserved in different geographic isolates of *L. donovani* and *L. infantum* (Joshi and Dwyer, 2007; Joshi et al., 2012). First, *L. donovani* promastigotes were observed to constitutively synthesize and release this nuclease, named LdNuc^s^, into their growth medium. Then, this activity was identified in axenic amastigotes as well as *in vivo*-derived amastigotes isolated from infected hamster spleen tissue (Joshi and Dwyer, 2007). Analyses of the LdNuc^s^-derived protein have confirmed the presence of the five conserved domains of class I nucleases (Joshi and Dwyer, 2007). Zymogram gels of cell lysates and culture supernatants showed marked differences between 3’-NT/NU and LdNuc^s^ activities. While 3’-NT/NU is ~43 kDa and insensitive to dithiothreitol (DTT) inhibition, LdNuc^s^ is a 35-kDa, DTT-sensitive nuclease. As previously described, 3’-NT/NU is a membrane-bound enzyme; therefore, its activity was detected only in cell lysates. On the other hand, LdNuc^s^ activity was detected in both cell lysates and concentrated cell-free culture supernatants (Joshi and Dwyer, 2007).

Coupled immunoprecipitation-enzymatic assays using epitope-tagged recombinant enzyme were performed to analyze other biochemical properties of LdNuc^s^ activity. The enzyme was capable of hydrolyzing RNA, single- and double-stranded DNA and a variety of synthetic polynucleotides. LdNuc^s^ appears to have a broad pH tolerance since nucleic acid hydrolysis occurred under both acidic (pH 5.0) and alkaline conditions (pH 8.5), suggesting that LdNuc^s^ may be completely functional within the different microenvironments faced by parasites during their lifecycle (Joshi and Dwyer, 2007).

Sera collected from infected visceral leishmaniasis patients from different geographic locations were able to recognize LdNuc^s,^ which clearly indicates that the enzyme is synthesized and expressed by *in vivo* amastigotes during the course of human infection (Joshi et al., 2012). The biological role played by LdNuc^s^ remains to be completely elucidated; however, the enzyme was postulated to participate in the purine salvage pathway. As a secreted protein, LdNuc^s^ can function far away from the parasite in the mobilization of host-derived nucleic acids. Together with 3’-NT/NU and other enzymes involved in purine salvage, LdNuc^s^ may contribute to the availability of extracellular nucleosides and nucleobases that can be uptaken by the parasite (Joshi and Dwyer, 2007) (**Figure 1B**).

## Concluding Remarks

Since the 1980s, with the first study describing the occurrence of 3’-NT/NU in *L. donovani*, several works have identified class I nucleases in different *Leishmania* species. Throughout this review, we discussed the occurrence and the major biochemical features of membrane-bound (3’-NT/NU), intracellular (P4/LmC1N), and secreted (LdNuc^s^) nucleases of this pathogenic parasite. The main properties and the proposed biological functions of these class I nucleases are summarized in **Table 1**. Beyond their different localization, these enzymes were also observed to display differential expression throughout the parasite lifecycle. While 3’-NT/NU is selectively expressed in promastigotes, P4/LmC1N and LdNuc^s^ predominated at amastigotes (Kar et al., 2000; Sopwith et al., 2002; Farajnia et al., 2004; Joshi and Dwyer, 2007; Lakhal-Naouar et al., 2008; Farajnia et al., 2011).

**Table 1:** Class I nucleases of *Leishmania* parasites.

Class I Nuclease	Expression during lifecycle	Localization	Potential physiological roles/applications	References
3’-NT/NU	Promastigote	Membrane-bound	Purine salvage pathway Purinergic signaling NETs hydrolysis	(Gottlieb, 1985; Debrabant et al., 1995; Lakhal-Naouar et al., 2008; Vieira et al., 2011; Guimarães-Costa et al., 2014)
P4/LmC1N	Amastigote	Perinuclear	Gene expression DNA repair Vaccine target	(Soong et al., 1995; Kar et al., 2000; Campbell et al., 2003; Farajnia et al., 2004; Fakhraee et al., 2016)
LdNuc^s^	Amastigote	Secreted	Purine salvage pathway	(Joshi and Dwyer, 2007; Joshi et al., 2012)

Due to its ecto-localization, 3’-NT/NU can hydrolyze extracellular substrates, which is also true for LdNuc^s^ because it is a secreted enzyme. Because of this feature, the first biological role proposed for these nucleases was their involvement in the purine acquisition process (Sopwith et al., 2002; Joshi and Dwyer, 2007; Lakhal-Naouar et al., 2008) (**Figures 1A, B****)**. In addition to the nutritional viewpoint, 3’-NT/NU has also been related to the establishment of parasite infection in mammalian hosts, as represented in **Figure 1A**. By hydrolyzing extracellular nucleic acids through its ecto-nuclease activity, *Leishmania* promastigotes can escape from NETs, thus avoiding a hostile microenvironment and being able to infect macrophages (Guimarães-Costa et al., 2014; Freitas-Mesquita et al., 2019). 3’-NT/NU can provide extracellular adenosine directly by 3’-AMP hydrolysis or by converting nucleic acids to 5’-monophosphorylated nucleotides, which culminates in adenosine generation by conjugate action with ecto-5’-nucleotidase. Adenosine can interact with purinergic receptors of the host immune system, favoring *Leishmania*-macrophage interactions (Paletta-Silva et al., 2011; Vieira et al., 2011; Paletta-Silva and Meyer-Fernandes, 2012; Freitas-Mesquita and Meyer-Fernandes, 2014; Freitas-Mesquita et al., 2016).

The roles played by 3’-NT/NU are significantly relevant at the early stages of infection, which is consistent with its expression in the promastigote form of the parasite. Metacyclic promastigotes are the infective form for mammalian hosts; thus, they face the first strategies of the immune system to impair the establishment of infection. However, parasites must differentiate into amastigotes to continue the infective process, subverting the killing repertoire of macrophages. The survival and proliferation of amastigotes inside the phagolysosome vacuole determine the success of parasite infection (Pace, 2014). In this context, enzymes important for amastigote development and survival are considered potential targets for chemotherapy against leishmaniasis. LdNuc^s^ is conserved in different geographical isolates of *L. donovani* and *L. infantum*, is expressed by *in vivo* amastigotes during the course of human infections, and is an interesting chemotherapeutic target due to its possible role in the purine salvage pathway (Joshi and Dwyer, 2007; Joshi et al., 2012).

Another nuclease expressed in amastigotes, named P4 nuclease, was first described in *L. pifanoi* and was shown to be located in the endoplasmic reticulum of the parasite. Due to its cellular location and its endonuclease activity, P4 nuclease is probably involved in mRNA stability and nucleotide excision and repair (**Figure 1B**). Homologs of P4 nuclease were described in several *Leishmania* species, including *L. major*, which was named LmC1N. By being a protein conserved among different species, P4/LmC1N emerges as an interesting target for the development of vaccines promoting cross-protection against different manifestations of leishmaniasis (Soong et al., 1995; Farajnia et al., 2005; Fakhraee et al., 2016). Although the results obtained thus far have suggested possible cross-protection triggered by the employment of P4/LmC1N antigens, different combinations of parasite genes and appropriate adjuvants must be investigated to reach optimal conditions.

Based on all data discussed throughout this review, class I nucleases seem to play different and important roles in parasite growth, survival, and development, including the establishment and maintenance of infection in mammalian hosts. It is noteworthy that more advanced approaches in the fields of molecular biology and bioinformatics would be crucial to provide more precise information about these enzymes. The silencing or overexpression of the genes encoding these proteins would be remarkably helpful to fully comprehend their physiological roles. The availability of the genome of different *Leishmania* species allows determining the structurally and functionally conservation of the class I nucleases throughout the parasites’ evolution. Additional studies are certainly required to deepen the knowledge about this important class of enzymes that has been considered a potential target for chemotherapy and prophylactics against leishmaniasis.

## Author Contributions

AF-M and JM-F wrote the manuscript. AF-M prepared the figure. All authors contributed to the article and approved the submitted version.

## Funding

This work was supported by grants from the Brazilian agencies Conselho Nacional de Desenvolvimento Científico e Tecnológico (CNPq - Grant Number: 401134/2014–8), Coordenação de Aperfeiçoamento de Pessoal de Nível superior (CAPES - Grant Number: 0012017) and Fundação Carlos Chagas Filho de Amparo à Pesquisa do Estado do Rio de Janeiro (FAPERJ - Grant Number: e-26/201.300/2014).

## Conflict of Interest

The authors declare that the research was conducted in the absence of any commercial or financial relationships that could be construed as a potential conflict of interest.

## Publisher’s Note

All claims expressed in this article are solely those of the authors and do not necessarily represent those of their affiliated organizations, or those of the publisher, the editors and the reviewers. Any product that may be evaluated in this article, or claim that may be made by its manufacturer, is not guaranteed or endorsed by the publisher.

## References

[B1] Afonso M. Mestre A. R. Silva G. Almeida A. C. Cunha R. A. Meyer-Fernandes J. R.. (2021). Candida Extracellular Nucleotide Metabolism Promotes Neutrophils Extracellular Traps Escape. Front. Cell. Infect. Microbiol. 11, 678568. doi: 10.3389/fcimb.2021.678568 34327150 PMC8313894

[B2] Ashutosh Sundar S. Goyal N. (2007). Molecular Mechanisms of Antimony Resistance in Leishmania. J. Med. Microbiol. 56 (2), 143–153. doi: 10.1099/jmm.0.46841-0 17244793

[B3] Bandyopadhyay R. Coutts M. Krowczynska A. Brawerman G. (1990). Nuclease Activity Associated With Mammalian mRNA in Its Native State: Possible Basis for Selectivity in mRNA Decay. Mol. Cell. Biol. 10 (5), 2060–2069. doi: 10.1128/mcb.10.5.2060-2069.1990 2325645 PMC360553

[B4] Bangs J. D. Brouch E. M. Ransom D. M. Roggy J. L. (1996). A Soluble Secretory Reporter System in Trypanosoma Brucei. Studies on Endoplasmic Reticulum Targeting. J. Biol. Chem. 271 (31), 18387–18393. doi: 10.1074/jbc.271.31.18387 8702482

[B5] Berends E. T. M. Horswill A. R. Haste N. M. Monestier M. Nizet V. von Köckritz-Blickwede M. (2010). Nuclease Expression by Staphylococcus Aureus Facilitates Escape From Neutrophil Extracellular Traps. J. Innate Immun. 2 (6), 576–586. doi: 10.1159/000319909 20829609 PMC2982853

[B6] Berman J. D. (1997). Human Leishmaniasis: Clinical, Diagnostic, and Chemotherapeutic Developments in the Last 10 Years. Clin. Infect. Dis. 24 (4), 684–703. doi: 10.1093/clind/24.4.684 9145744

[B7] Borja-Cabrera G. P. Santos F. B. Picillo E. Gravino A. E. Manna L. Palatnik-de-Sousa C. B. (2009). Nucleoside Hydrolase DNA Vaccine Against Canine Visceral Leishmaniasis. Proc. Vaccinol 1 (1), 104–109. doi: 10.1016/j.provac.2009.07.019 PMC7129871 32288909

[B8] Brinkmann V. Reichard U. Goosmann C. Fauler B. Uhlemann Y. Weiss D. S.. (2004). Neutrophil Extracellular Traps Kill Bacteria. Sci. (New York N.Y.) 303 (5663), 1532–1535. doi: 10.1126/science.1092385 15001782

[B9] Campbell K. Diao H. Ji J. Soong L. (2003). DNA Immunization With the Gene Encoding P4 Nuclease of Leishmania Amazonensis Protects Mice Against Cutaneous Leishmaniasis. Infect. Immun. 71 (11), 6270–6278. doi: 10.1128/IAI.71.11.6270-6278.2003 14573646 PMC219588

[B10] Campbell T. A. Zlotnick G. W. Neubert T. A. Sacci J. B. Gottlieb M. (1991). Purification and Characterization of the 3’-Nucleotidase/Nuclease From Promastigotes of Leishmania Donovani. Mol. Biochem. Parasitol. 47 (1), 109–117. doi: 10.1016/0166-6851(91)90153-w 1857379

[B11] Chang K. P. (1983). Cellular and Molecular Mechanisms of Intracellular Symbiosis in Leishmaniasis. Int. Rev. Cytology. Supplement 14, 267–305. 6341275

[B12] Coler R. Reed S. (2005). Second-Generation Vaccines Against Leishmaniasis. Trends Parasitol 21 (5), 244–249. doi: 10.1016/j.pt.2005.03.006 15837614

[B13] Croft S. L. Olliaro P. (2011). Leishmaniasis Chemotherapy—Challenges and Opportunities. Clin. Microbiol. Infection 17 (10), 1478–1483. doi: 10.1111/j.1469-0691.2011.03630.x 21933306

[B14] Debrabant A. Ghedin E. Dwyer D. M. (2000). Dissection of the Functional Domains of the Leishmania Surface Membrane 3’-Nucleotidase/Nuclease, a Unique Member of the Class I Nuclease Family. J. Biol. Chem. 275 (21), 16366–16372. doi: 10.1074/jbc.M908725199 10748102

[B15] Debrabant A. Gottlieb M. Dwyer D. M. (1995). Isolation and Characterization of the Gene Encoding the Surface Membrane 3’-Nucleotidase/Nuclease of Leishmania Donovani. Mol. Biochem. Parasitol. 71 (1), 51–63. doi: 10.1016/0166-6851(95)00035-y 7630383

[B16] De Oliveira M. C. Boutet V. Fattal E. Boquet D. Grognet J. M. Couvreur P.. (2000). Improvement of *In Vivo* Stability of Phosphodiester Oligonucleotide Using Anionic Liposomes in Mice. Life Sci. 67 (13), 1625–1637. doi: 10.1016/s0024-3205(00)00745-1 10983856

[B17] Desai N. A. Shankar V. (2003). Single-Strand-Specific Nucleases. FEMS Microbiol. Rev. 26 (5), 457–491. doi: 10.1111/j.1574-6976.2003.tb00626.x 12586391

[B18] Dunning N. (2009). Leishmania Vaccines: From Leishmanization to the Era of DNA Technology. Bioscience Horizons 2 (1), 73–82. doi: 10.1093/biohorizons/hzp004

[B19] Fakhraee F. Badiee A. Alavizadeh S. H. Jalali S. A. Chavoshian O. Khamesipour A.. (2016). Coadminstration of L. Major Amastigote Class I Nuclease (Rlmacin) With LPD Nanoparticles Delays the Progression of Skin Lesion and the L. Major Dissemination to the Spleen in BALB/c Mice-Based Experimental Setting. Acta Tropica 159, 211–218. doi: 10.1016/j.actatropica.2016.04.004 27060774

[B20] Farajnia S. Alimohammadian M. H. Reiner N. E. Karimi M. Ajdari S. Mahboudi F. (2004). Molecular Characterization of a Novel Amastigote Stage Specific Class I Nuclease From Leishmania Major. Int. J. Parasitol 34 (8), 899–908. doi: 10.1016/j.ijpara.2004.03.005 15217728

[B21] Farajnia S. Mahboudi F. Ajdari S. Reiner N. E. Kariminia A. Alimohammadian M. H. (2005). Mononuclear Cells From Patients Recovered From Cutaneous Leishmaniasis Respond to Leishmania Major Amastigote Class I Nuclease With a Predominant Th1-Like Response. Clin. Exp. Immunol. 139 (3), 498–505. doi: 10.1111/j.1365-2249.2004.02702.x 15730396 PMC1809324

[B22] Farajnia S. Rahbarnia L. Maleki Zanjani B. Alimohammadian M. H. Abdoli Oskoee S. Beh-Pajooh A.. (2011). Molecular Cloning and Characterization of P4 Nuclease From Leishmania Infantum. Enzyme Res. 2011 (1), 1–6. doi: 10.4061/2011/970983 PMC3132502 21755045

[B23] Freitas-Mesquita A. L. Dick C. F. Dos-Santos A. L. A. Nascimento M. T. C. Rochael N. C. Saraiva E. M.. (2019). Cloning, Expression and Purification of 3’-Nucleotidase/Nuclease, an Enzyme Responsible for the Leishmania Escape From Neutrophil Extracellular Traps. Mol. Biochem. Parasitol 229, 6–14. doi: 10.1016/j.molbiopara.2019.02.004 30772424

[B24] Freitas-Mesquita A. L. Gomes M. T. Vieira D. P. Paes-Vieira L. Nascimento M. T. C. Lopes A. H. C. S.. (2016). Inhibitory Effects Promoted by 5′-Nucleotides on the Ecto-3′-Nucleotidase Activity of Leishmania Amazonensis. Exp. Parasitol 169, 111–118. doi: 10.1016/j.exppara.2016.08.001 27531705

[B25] Freitas-Mesquita A. L. Meyer-Fernandes J. R. (2014). Ecto-Nucleotidases and Ecto-Phosphatases From Leishmania and Trypanosoma Parasites. Sub-Cellular Biochem. 74, 217–252. doi: 10.1007/978-94-007-7305-9_10 24264248

[B26] Freitas-Mesquita A. L. Meyer-Fernandes J. R. (2017). 3′Nucleotidase/Nuclease in Protozoan Parasites: Molecular and Biochemical Properties and Physiological Roles. Exp. Parasitol 179, 1–6. doi: 10.1016/j.exppara.2017.06.001 28587841

[B27] Gottlieb M. (1985). Enzyme Regulation in a Trypanosomatid: Effect of Purine Starvation on Levels of 3’-Nucleotidase Activity. Sci. (New York N.Y.) 227 (4682), 72–74. doi: 10.1126/science.2981117 2981117

[B28] Gottlieb M. (1989). The Surface Membrane 3’-Nucleotidase/Nuclease of Trypanosomatid Protozoa. Parasitol. Today (Personal Ed.) 5 (8), 257–260. doi: 10.1016/0169-4758(89)90259-7 15463228

[B29] Gottlieb M. Dwyer D. M. (1981). Leishmania Donovani: Surface Membrane Acid Phosphatase Activity of Promastigotes. Exp. Parasitol. 52 (1), 117–128. doi: 10.1016/0014-4894(81)90067-9 7238722

[B30] Gottlieb M. Dwyer D. (1983). Evidence for Distinct 5’- and 3’-Nucleotidase Activities in the Surface Membrane Fraction of Leishmania Donovani Promastigotes. Mol. Biochem. Parasitol 7 (4), 303–317. doi: 10.1016/0166-6851(83)90013-0 6308442

[B31] Greenblatt C. L. (1980). The Present and Future of Vaccination for Cutaneous Leishmaniasis. Prog. Clin. Biol. Res. 47, 259–285. 7010374

[B32] Guimarães-Costa A. B. DeSouza-Vieira T. S. Paletta-Silva R. Freitas-Mesquita A. L. Meyer-Fernandes J. R. Saraiva E. M. (2014). 3’-Nucleotidase/Nuclease Activity Allows Leishmania Parasites to Escape Killing by Neutrophil Extracellular Traps. Infection Immun. 82 (4), 1732–1740. doi: 10.1128/IAI.01232-13 PMC3993383 24516114

[B33] Guimarães-Costa A. B. Nascimento M. T. C. Wardini A. B. Pinto-da-Silva L. H. Saraiva E. M. (2012). ETosis: A Microbicidal Mechanism Beyond Cell Death. J. Parasitol Res. 2012:929743. doi: 10.1155/2012/929743 22536481 PMC3321301

[B34] Hammond D. J. Gutteridge W. E. (1984). Purine and Pyrimidine Metabolism in the Trypanosomatidae. Mol. Biochem. Parasitol 13 (3), 243–261. doi: 10.1016/0166-6851(84)90117-8 6396514

[B35] Hassan H. F. Coombs G. H. (1987). Phosphomonoesterases of Leishmania Mexicana Mexicana and Other Flagellates. Mol. Biochem. Parasitol 23 (3), 285–296. doi: 10.1016/0166-6851(87)90035-1 3037369

[B36] Herwaldt B. L. Berman J. D. (1992). Recommendations for Treating Leishmaniasis With Sodium Stibogluconate (Pentostam) and Review of Pertinent Clinical Studies. Am. J. Trop. Med. Hygiene 46 (3), 296–306. doi: 10.4269/ajtmh.1992.46.296 1313656

[B37] Joshi M. B. Dwyer D. M. (2007). Molecular and Functional Analyses of a Novel Class I Secretory Nuclease From the Human Pathogen, Leishmania Donovani. J. Biol. Chem. 282 (13), 10079–10095. doi: 10.1074/jbc.M610770200 17276983

[B38] Joshi M. B. Hernandez Y. Owings J. P. Dwyer D. M. (2012). Diverse Viscerotropic Isolates of Leishmania All Express a Highly Conserved Secretory Nuclease During Human Infections. Mol. Cell. Biochem. 361 (1–2), 169–179. doi: 10.1007/s11010-011-1101-1 22020747 PMC3706101

[B39] Kar S. Soong L. Colmenares M. Goldsmith-Pestana K. McMahon-Pratt D. (2000). The Immunologically Protective P-4 Antigen of Leishmania Amastigotes. A Developmentally Regulated Single Strand-Specific Nuclease Associated With the Endoplasmic Reticulum. J. Biol. Chem. 275 (48), 37789–37797. doi: 10.1074/jbc.M002149200 10969068

[B40] Lakhal-Naouar I. Ben Achour-Chenik Y. Boublik Y. Meddeb M. Aamouri A. Fattoum A.. (2008). Identification and Characterization of a New Leishmania Major Specific 3’nucleotidase/Nuclease Protein. Biochem. Biophys. Res. Commun. 375 (1), 54–58. doi: 10.1016/j.bbrc.2008.07.099 18674514

[B41] Mattner F. Magram J. Ferrante J. Launois P. Di Padova K. Behin R.. (1996). Genetically Resistant Mice Lacking Interleukin-12 Are Susceptible to Infection With Leishmania Major and Mount a Polarized Th2 Cell Response. Eur. J. Immunol. 26 (7), 1553–1559. doi: 10.1002/eji.1830260722 8766560

[B42] Miura R. Kooriyama T. Yoneda M. Takenaka A. Doki M. Goto Y.. (2015). Efficacy of Recombinant Canine Distemper Virus Expressing Leishmania Antigen Against Leishmania Challenge in Dogs. PloS Negl. Trop. Dis. 9 (7), e0003914. doi: 10.1371/journal.pntd.0003914 26162094 PMC4498809

[B43] Modabber F. (1995). Vaccines Against Leishmaniasis. Ann. Trop. Med. Parasitol. 89 Suppl 1, 83–88. doi: 10.1080/00034983.1995.11813017 8745930

[B44] Moreno J. Vouldoukis I. Schreiber P. Martin V. McGahie D. Gueguen S.. (2014). Primary Vaccination With the LiESP/QA-21 Vaccine (CaniLeish) Produces a Cell-Mediated Immune Response Which Is Still Present 1 Year Later. Veterinary Immunol. Immunopathol 158 (3–4), 199–207. doi: 10.1016/j.vetimm.2014.01.011 24560650

[B45] Neubert T. A. Gottlieb M. (1990). An Inducible 3’-Nucleotidase/Nuclease From the Trypanosomatid Crithidia Luciliae. Purification and Characterization. J. Biol. Chem. 265 (13), 7236–7242. 2158995

[B46] Pace D. (2014). Leishmaniasis. J. Infection 69, S10–S18. doi: 10.1016/j.jinf.2014.07.016 25238669

[B47] Palatnik-de-Sousa C. B. Barbosa A. de F. Oliveira S. M. Nico D. Bernardo R. R.. (2008). FML Vaccine Against Canine Visceral Leishmaniasis: From Second-Generation to Synthetic Vaccine. Expert Rev. Vaccines 7 (6), 833–851. doi: 10.1586/14760584.7.6.833 18665780

[B48] Paletta-Silva R. Meyer-Fernandes J. R. (2012). Adenosine and Immune Imbalance in Visceral Leishmaniasis: The Possible Role of Ectonucleotidases. J. Trop. Med. 2012, 1–6. doi: 10.1155/2012/650874 PMC3189589 22007242

[B49] Paletta-Silva R. Vieira D. P. Vieira-Bernardo R. Majerowicz D. Gondim K. C. Vannier-Santos M. A.. (2011). Leishmania Amazonensis: Characterization of an Ecto-3’-Nucleotidase Activity and Its Possible Role in Virulence. Exp. Parasitol 129 (3), 277–283. doi: 10.1016/j.exppara.2011.07.014 21827749

[B50] Podinovskaia M. Descoteaux A. (2015). Leishmania and the Macrophage: A Multifaceted Interaction. Future Microbiol. 10 (1), 111–129. doi: 10.2217/fmb.14.103 25598341

[B51] Rachamim N. Jaffe C. L. (1993). Pure Protein From Leishmania Donovani Protects Mice Against Both Cutaneous and Visceral Leishmaniasis. J. Immunol. (Baltimore Md.: 1950) 150 (6), 2322–2331. 8450215

[B52] Romier C. Dominguez R. Lahm A. Dahl O. Suck D. (1998). Recognition of Single-Stranded DNA by Nuclease P1: High Resolution Crystal Structures of Complexes With Substrate Analogs. Proteins 32 (4), 414–424. doi: 10.1002/(SICI)1097-0134(19980901)32:4<414::AID-PROT2>3.0.CO;2-G 9726413

[B53] Ross J. (1996). Control of Messenger RNA Stability in Higher Eukaryotes. Trends Genetics: TIG 12 (5), 171–175. doi: 10.1016/0168-9525(96)10016-0 8984731

[B54] Sacci J. B. Campbell T. A. Gottlieb M. (1990). Leishmania Donovani: Regulated Changes in the Level of Expression of the Surface 3’-Nucleotidase/Nuclease. Exp. Parasitol 71 (2), 158–168. doi: 10.1016/0014-4894(90)90018-8 2164952

[B55] Seper A. Hosseinzadeh A. Gorkiewicz G. Lichtenegger S. Roier S. Leitner D. R.. (2013). Vibrio Cholerae Evades Neutrophil Extracellular Traps by the Activity of Two Extracellular Nucleases. PloS Pathog. 9 (9), e1003614. doi: 10.1371/journal.ppat.1003614 24039581 PMC3764145

[B56] Singh B. Sundar S. (2012). Leishmaniasis: Vaccine Candidates and Perspectives. Vaccine 30 (26), 3834–3842. doi: 10.1016/j.vaccine.2012.03.068 22475861

[B57] Smooker P. M. Rainczuk A. Kennedy N. Spithill T. W. (2004). DNA Vaccines and Their Application Against Parasites–Promise, Limitations and Potential Solutions. Biotechnol. Annu. Rev. 10, 189–236. doi: 10.1016/S1387-2656(04)10007-0 15504707

[B58] Soong L. Duboise S. M. Kima P. McMahon-Pratt D. (1995). Leishmania Pifanoi Amastigote Antigens Protect Mice Against Cutaneous Leishmaniasis. Infection Immun. 63 (9), 3559–3566. doi: 10.1128/iai.63.9.3559-3566.1995 PMC173494 7642292

[B59] Sopwith W. F. Debrabant A. Yamage M. Dwyer D. M. Bates P. A. (2002). Developmentally Regulated Expression of a Cell Surface Class I Nuclease in Leishmania Mexicana. Int. J. Parasitol. 32 (4), 449–459. doi: 10.1016/s0020-7519(01)00372-1 11849641

[B60] Stober C. B. Lange U. G. Roberts M. T. M. Gilmartin B. Francis R. Almeida R.. (2006). From Genome to Vaccines for Leishmaniasis: Screening 100 Novel Vaccine Candidates Against Murine Leishmania Major Infection. Vaccine 24 (14), 2602–2616. doi: 10.1016/j.vaccine.2005.12.012 16406227

[B61] Sumby P. Barbian K. D. Gardner D. J. Whitney A. R. Welty D. M. Long R. D.. (2005). Extracellular Deoxyribonuclease Made by Group A Streptococcus Assists Pathogenesis by Enhancing Evasion of the Innate Immune Response. Proc. Natl. Acad. Sci. U. S. A. 102 (5), 1679–1684. doi: 10.1073/pnas.0406641102 15668390 PMC547841

[B62] Thammavongsa V. Missiakas D. M. Schneewind O. (2013). Staphylococcus Aureus Degrades Neutrophil Extracellular Traps to Promote Immune Cell Death. Sci. (New York N.Y.) 342 (6160), 863–866. doi: 10.1126/science.1242255 PMC4026193 24233725

[B63] Vannier-Santos M. Martiny A. Souza W. (2002). Cell Biology of Leishmania Spp.: Invading and Evading. Curr. Pharm. Design 8 (4), 297–318. doi: 10.2174/1381612023396230 11860368

[B64] Vieira D. P. Paletta-Silva R. Saraiva E. M. Lopes A. H. C. S. Meyer-Fernandes J. R. (2011). Leishmania Chagasi: An Ecto-3’-Nucleotidase Activity Modulated by Inorganic Phosphate and Its Possible Involvement in Parasite-Macrophage Interaction. Exp. Parasitol 127 (3), 702–707. doi: 10.1016/j.exppara.2010.11.003 21111737

[B65] Volbeda A. Lahm A. Sakiyama F. Suck D. (1991). Crystal Structure of Penicillium Citrinum P1 Nuclease at 2.8 A Resolution. EMBO J. 10 (7), 1607–1618. 1710977 10.1002/j.1460-2075.1991.tb07683.x PMC452829

[B66] Wang Y. Kelly C. G. Singh M. McGowan E. G. Carrara A.-S. Bergmeier L. A.. (2002). Stimulation of Th1-Polarizing Cytokines, C-C Chemokines, Maturation of Dendritic Cells, and Adjuvant Function by the Peptide Binding Fragment of Heat Shock Protein 70. J. Immunol. (Baltimore Md.: 1950) 169 (5), 2422–2429. doi: 10.4049/jimmunol.169.5.2422 12193710

[B67] Wilson C. M. (1982). Plant Nucleases: Biochemistry and Development of Multiple Molecular Forms. Isozymes 6, 33–54. 6299997

[B68] Yamage M. Debrabant A. Dwyer D. M. (2000). Molecular Characterization of a Hyperinducible, Surface Membrane-Anchored, Class I Nuclease of a Trypanosomatid Parasite. J. Biol. Chem. 275 (46), 36369–36379. doi: 10.1074/jbc.M004036200 10945983

